# Mechanical Properties and Energy Absorption Characteristics of a Combined Gradient BCC Lattice Structure: A Numerical Study

**DOI:** 10.3390/ma18153652

**Published:** 2025-08-04

**Authors:** Xiangheng Zhao, Xiaoqiang Wang, Yunmiao Shang

**Affiliations:** 1 International Education College, Shenyang Aerospace University, Shenyang 110136, China; zhaoxiangheng@stu.sau.edu.cn (X.Z.); shangyunmiao@stu.sau.edu.cn (Y.S.); 2 Aeronautical and Astronautical College, Shenyang Aerospace University, Shenyang 110136, China

**Keywords:** lattice structure, relative density gradient, mechanical performance, energy absorption

## Abstract

As a new functional graded lattice structure construction strategy, the relative density gradient strategy has a promising future due to its ease of realization in various lattice structures. This paper proposes a BCC lattice structure combining two different lattice single cells. Based on this, the single cells of different structures are assigned different relative density gradients, resulting in 18 combined gradient lattice structures. Based on proving the experimental feasibility of numerical simulation, the mechanical properties and energy absorption characteristics of the combined gradient lattice structure are investigated by numerical simulation. When applied to composite lattice structures, the proposed wave-like gradient design significantly improves mechanical properties. Among the various gradient strategies examined, several have achieved mechanical performance close to that of uniform lattice structures. To some extent, this approach mitigates the common drawback of gradient lattice structures—where the relative density of the weakest layer is consistently lower than the interlayer relative density of uniform lattice structures—resulting in varying degrees of mechanical performance degradation compared to their uniform counterparts. The proposed linearly enhanced gradient strategy (Strategy-LE) possesses higher SEA and CLE values when the lattice structure is subjected to compressive loading, with an improvement of 6.36% in SEA and 61.6% in CLE over the uniform structure. Through the relative density gradient design, the adaptability of the BCC lattice structure in actual complex application scenarios is greatly enhanced, and the energy-absorbing properties of the lattice structure are greatly improved.

## 1. Introduction

Mechanical metamaterials are new materials that can be artificially designed and constructed to achieve novel properties that cannot be achieved with natural materials, such as high specific strength and stiffness [1,2,3], excellent energy absorption properties [4], reversible shape deformation [5], sound absorption and insulation [6], etc. With the rapid development of aerospace, biomedical, construction engineering, rail transportation, and other fields, the demand for lightweight high-performance materials is increasing [7,8,9,10,11,12]. Therefore, mechanical metamaterials [13,14,15] have received extensive attention in the research field.

Lightweight and high-strength lattice-like structure [4,16,17,18,19] is a significant branch of mechanical metamaterials that utilizes the compositional characteristics of nature’s crystal structure as a guideline for construction and arranges the well-designed single cells in regular repetitions to form a lattice structure. This approach forms a new type of material under this rule. Therefore, compared with traditional irregular materials, the lattice structure has significant advantages in wide designability, tunable properties, and outstanding multi-functionality, enabling it to stand out among numerous mechanical metamaterials.

With the same materials used for fabrication, the different topologies of single cells affect the mechanical properties of the lattice structure. Therefore, the design problem of single-cell structure has become a significant research hotspot. Based on the current findings of single-cell structures, researchers have categorized the lattice structures into tensile or bending-dominated lattice structures [20,21,22], lattice structures associated with BCC [23,24,25]/FCC [26,27], chiral/anti-chiral geometry structures [28,29,30,31], etc. Among them, lattice structures associated with BCC/FCC stand out from many categories due to their simple construction, high porosity, light weight, and high strength. Most of the current research [32] focuses only on the design of the single-cell structure, which is then only replicated regularly to form the lattice structure. It is not conducive to the filling of a large number of irregularly shaped components in practical application scenarios. Currently, it is necessary to investigate more lattice structure building methods to generate filling strategies that are more adaptable to the shape of the components.

As a novel structure construction strategy, functionally graded lattice structure has been widely used in recent studies due to its more versatile designability than traditional construction methods and easier application in more complex real-world scenarios [33,34,35,36,37,38,39]. Functionally graded lattice structure subtly alters the properties of the overall structure by adjusting the properties of some of the single cells in the overall lattice structure. The tuning methods can be broadly categorized into two main groups: (1) Using a single single-cell type to construct the lattice structure by varying the porosity between cells or setting up a relative density gradient between cells [40]. (2) Using two or more single-cell types to construct the lattice structure by pairing several types of single cells in a design [41].

Among them, the relative density gradient strategy is very advantageous. It has a promising future due to its straightforward design concept, making it easier to realize in various types of lattice structures. The current relative density gradient strategies are mainly designed in the longitudinal direction (compression direction in experiments) of a lattice structure composed of one type of single cell, with linear relative density or linear symmetric relative density [40]. However, the relative density design remains in lattice structures containing only one single-cell type. The study of combinatorial gradient design strategies for lattice structures consisting of only one class of single-cell but possessing two or even more single-cell types, or lattice structures containing two or even more classes of single cells (e.g., consisting of both BCCs and FCCs [41]) has rarely been reported in the literature. Therefore, it is crucial to study the mechanical properties of lattice structures with more complex relative density gradients in conjunction with the design concept of combinatorial gradients to explore the application of gradient lattice structures in practical and more complicated scenarios.

Based on the most basic BCC single cell structure composed of multiple symmetrical cylindrical rods, this paper proposes a “wave-like” gradient design strategy. Combined with the existing three density gradient design strategies, different gradient designs are assigned to eight and four cylindrical support rods in the designed composite lattice structure. Several composite gradient lattice structures are constructed. For each of the various combined gradient lattice structures formed, the J-C constitutive model of the AlSi10Mg material was used to conduct finite element simulation experiments to investigate their mechanical properties and energy absorption characteristics.

In Section 2 (Design Architecture for Combined Gradient Lattice Structures), the lattice structure is named, and the gradient’s design is described. In Section 3 (Results and Discussion), the steps of the numerical simulation experiment are first described. Then the simulation results’ mechanical properties (Mises stress contour plots and stress–strain curves) and energy absorption characteristics (SEA and CLE values) are discussed separately.

Among them, the accuracy of the numerical model is verified through the quasi-static compression numerical simulation experiment of a 5 × 5 × 5 uniform, regular tetrahedral BCC lattice structure in Section 4.1. By carefully adjusting the relevant parameters of the numerical model, the simulation results are compared and fitted with the stress–strain curves obtained from experiments and numerical simulations under the same conditions in the literature. The good fitting results verify the feasibility of the numerical model and serve as the basis for the finite element simulation experiments of the lattice structure with combined gradients. This method can effectively ensure the accuracy of the numerical simulation experiments.

## 2. Design Architecture for Combined Gradient Lattice Structures

### 2.1. Naming Convention

**Figure 1:**
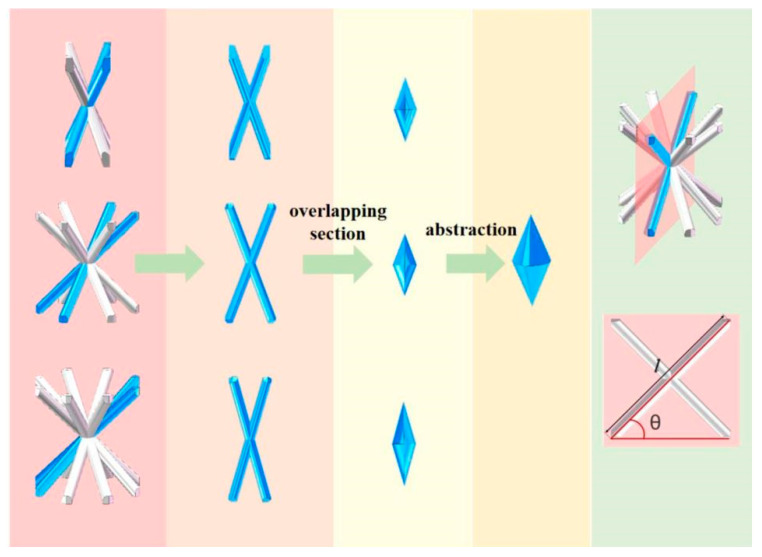
Simplification of the overlap portion between two neighboring rods in a single cell.

As shown in Figure 1, an ordinary BCC single-cell structure is formed by n tilted cylindrical rods with the same radius and angle with respect to the horizontal plane, which pass through its geometric center point together. An adjustable value of n varies the topology of the single cell. For simplicity, this single cell structure is named “SCn”, where “n” denotes the number of identical cylindrical rods forming the single cell, and "SC" is the abbreviation of “single cell”. 

### 2.2. Relative Density

The geometrical significance of relative density in BCC single-cell structure is the ratio of the total volume of all the struts in the cell to the volume occupied by the space cell, denoted by “ρ”. The relative density greatly influences the mechanical properties of the lattice structure. The relative density of a single cell is calculated as

(1) $$\rho_{SC}=\frac{V_{\mathrm{cylindricity}}}{V_{\mathrm{space}\;\mathrm{volume}}}$$

Among them, V_cylindricity_ and V_space volume_ are the total volume of cylindrical rods within the single cell and the volume occupied by the single cell in space, respectively. To calculate the relative density of the lattice cell more accurately, the first step is to calculate the total volume of the cylindrical rods within the cell. Due to the non-negligible overlap between the cylindrical rods, it is impossible to calculate the total volume of the rods by simply summing up the volumes of the individual rods before the overlap. By using commercial software SOLIDWORKS 2023, it was found that different “n” values of the single cell (SC4, SC6, SC8) in the shape of the overlap between neighboring rods are similar to each other, based on the two symmetric upper and lower symmetric cone combinations, as shown in Figure 1. On this basis, the formula for the relative density of single cells with different values of “n” is obtained as follows [40]:

(2) $$\rho_{SC}=\frac{V_{\mathrm{cylindricity}}}{V_{\mathrm{space}\;\mathrm{volume}}}=\frac{8\pi \left[nr^{2}l-\frac{(n-1){\left(\frac{r}{\sin\theta }\right)}^{3}}{3\tan[\frac{1}{2}[\arccos[1-2\cos^{2}\theta \sin^{2}(\frac{\pi }{n})]]]}\right]}{nl^{3}\sin\theta \cos^{2}\theta \sin(\frac{2\pi }{n})}$$

where l is the length of an inclined strut in a single cell, and θ is the angle between the inclined strut and the horizontal plane in a single cell, as shown in Figure 1.

The formula was used to calculate the relative densities of single cells’ different “n” values (SC4, SC6, SC8). CAD software determined the relative densities of the selected single cells under the same conditions. The relative error was calculated using the relative error formula Equation (3). It was verified that the relative error was less than 3%, and the accuracy of the formula Equation (2) calculation was good.

(3) $$\delta_{SC}=\left|\frac{\rho_{\mathrm{Calculated}\;\mathrm{SC}}-\rho_{\mathrm{Real}\;\mathrm{SC}}}{\rho_{\mathrm{Real}\;\mathrm{SC}}}\right|\times 100\%$$

### 2.3. The Design and Construction of the Combined Gradient Lattice Structure

#### 2.3.1. Combined Lattice Structure Consisting of Two Single-Cell Types

A lattice structure is a lattice-like dense row of countless single-cell structures formed in three dimensions by combining them in simpler repetitions.

As mentioned earlier, most relative density designs use lattice structures containing only one type of cell, i.e., a lattice structure containing only a single cell with a single “n” value. This design strategy allows the common “n” values of 3, 4, and 6 (SC3, SC4, and SC6) to be seamlessly and perfectly combined to form a lattice structure with a specific single-cell topology.

**Figure 2:**
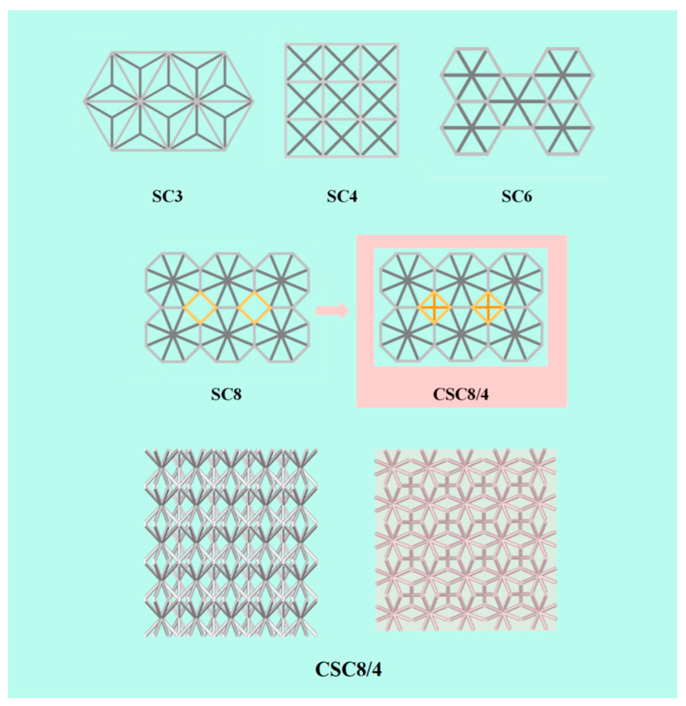
Design of the combined lattice structure.

However, when the value of “n” grows from 6 to 8, the problem of intercellular splicing begins to appear. A rectangular gap with square top and bottom surfaces is formed between the single-cell SC8s, as shown in Figure 2, which is not conducive to enhancing the stability of the lattice structure and its mechanical properties. By observing the characteristics of the voids between the single-cell SC8s, this paper proposes a design strategy using well-designed SC4 cells for void filling, which perfectly eliminates the performance defects that the larger voids may bring. The multilayer lattice structure formed by two different single cells is named the combined lattice structure, which is represented by the symbol “CSCn/n′,” and the design of CSC8/4 is shown in Figure 2.

This paper investigates the proposed CSC8/4 combinatorial structure to establish a 5 × 5 × 5 combinatorial lattice structure, where “5 × 5” in the first place represents the number of SC8 single cells in each layer, and “5” in the last place represents that the number of layers of the lattice structure is 5. Under this condition, each layer of the combined single-cell structure contains “4 × 4” SC4 single cells. The heights of the single cells are all 10 mm, i.e., the height of all lattice structures is 50 mm.

#### 2.3.2. Design of Combined Gradient Lattice Structures

**Figure 3:**
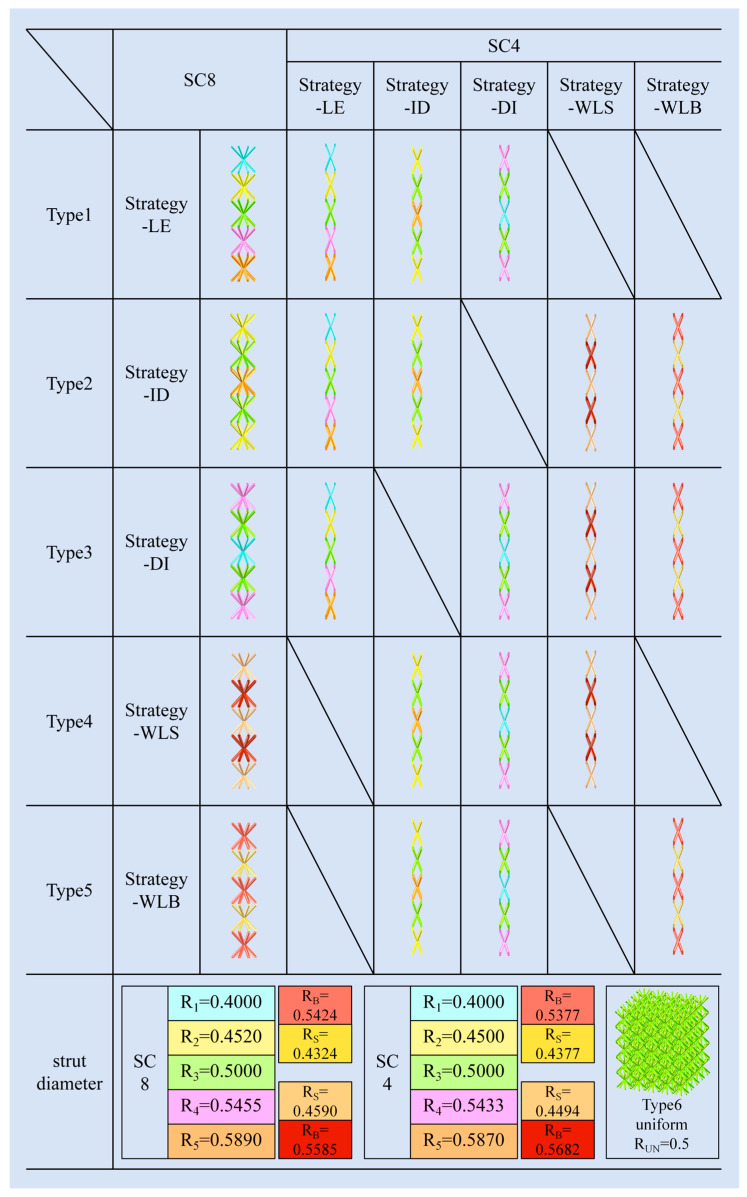
Lattice structure gradient strategy.

The gradient distribution proposed in this paper is based on the relative density gradient. Therefore, in a multilayer lattice single-cell structure, keeping the spatial dimensions of the single cell unchanged, the diameter of the cylindrical rods in the single cell is regularly varied, so that the combined gradient lattice structure formed under the same geometrical configuration possesses the same overall relative density as that of the uniform lattice structure. In this paper, we keep the same diameter of the single-cell stubs in each layer of the same configuration for the design, and adjust the relative density difference between the layers. The cylindrical rod diameter design of SC8 single cells is used as a benchmark to categorize the proposed relative density design strategies. A total of 17 relative density design strategies with different laws were proposed for five categories, as shown in Figure 3. The relative density gradient design under all laws contains four gradient construction strategies, which are the “wave-like” gradient strategy, linearly enhanced gradient strategy, increase add decrease gradient strategy, and decrease add increase gradient strategy. They are named “Strategy-WL”, “Strategy-LE (linearly enhanced)”, “Strategy-ID”, and “Strategy-DI”.

In this paper, based on the existing gradient design strategy, a new “wave-like” gradient design strategy is proposed to form a wave-like fluctuating gradient between the layers of the lattice structure by, for example, decreasing, then increasing, then decreasing the diameter of the single cells again in the layer. The specific naming background takes the example of the total number of layers of the lattice structure as an odd number of layers. When the single cells in the top and bottom layers have small diameters, the gradient strategy is named the “small wave-like” gradient strategy (Strategy-WLS). Similarly, when the top and bottom single cells have large diameters, the gradient strategy is named the “big wave-like” gradient strategy (Strategy-WLB). The “linearly enhanced” gradient strategy (Strategy-LE) creates a gradient design by increasing the diameter of single cells layer by layer from the top to the bottom of the lattice structure. The “decrease add increase” and “increase add decrease” gradient strategies treat the lattice structure as symmetric configurations about the middle layer, increasing or decreasing the diameter of the single cells from the top layer to the middle layer, respectively.

The combined relative density gradient strategies are categorized into five types, based on the principle of whether or not they have a similarity of gradient design, as shown in Figure 3. Type1 is classified based on the SC8-linearly enhanced gradient strategy, and combined with the SC8 single cells, respectively, are three sets of SC4 single cells with different gradient designs: the SC4-Strategy-LE, the SC4-Strategy-ID, and the SC4-Strategy-DI. Type2 uses the SC8-increase add decrease gradient strategy as a baseline, and combines SC8-Strategy-ID with SC4-Strategy-ID, SC4-Strategy-LE, SC4-Strategy-WLB, and SC4-Strategy-WLS, respectively, to form four kinds of gradient designs. Type3 also contains four strategies, combining the SC8-Strategy-DI with the SC4 single-cell “Strategy-DI”, “Strategy-WLB”, “Strategy-WLS”, and “Strategy-LE” relative density gradient design methods. The SC8 cells of Type4 and Type5 adopt “wave-like” gradient strategies, Strategy-WLB, and Strategy-WLS, respectively. The SC8 cells in the two types are combined with the SC4 cells that have the same gradient design strategy to form one design scheme, and then combined with the “Strategy-ID” and “Strategy-DI” schemes of SC4 cells to form three combinations in each type. The SC8 cells in the two types are combined with the SC4 cells with the same gradient design strategy to create a design scheme, and then combined with the “Strategy-ID” and “Strategy-DI” schemes of the SC4 cells to form three combined gradient designs in each type.

The relative density distributions of the schemes and the radii of the cylindrical rods within the various SC8 and SC4 single cells are distinguished by different colors, and the specific values of their radii are also shown in Figure 3. Among them, Type6 is a uniform lattice structure without gradients, and the radius parameters are also shown together.

Through careful adjustment, R_1_~R_5_ cleverly contain all the single-cell cylindrical rod radii used in the Strategy-LE, Strategy-ID, and Strategy-DI schemes, avoiding the clutter of radius parameters as much as possible. Based on the five-layer structure design of the lattice structure, the same radii are used for SC8 and SC4 cells of the same number of layers, respectively. The Strategy-LE scheme sequentially adopts the radii of cylindrical rods from R1 to R5 from the top to the bottom.

The Strategy-ID scheme uses different interlayer cylindrical rod radii of R_2_, R_3_, R_5_, R_3_, and R_2_ in sequence based on its gradient change method of increasing and then decreasing. Similarly, based on the gradient change in decreasing and then increasing, the cylindrical rod radii of the Strategy-DI scheme are R4, R3, R1, R3, and R_4_ from the top layer to the bottom layer, respectively. In the Strategy-WL scheme, R_B_ denotes the larger cylindrical rod radius and R_S_ denotes the smaller one. In this scheme, the lattice structure uses the same radius for layers 1, 3, and 5 from top to bottom, and the same radius for layers 2 and 4.

## 3. Finite Element Modeling Setup

The effect of relative density distribution on the mechanical properties and deformation mechanism of multilayer lattice structures under quasi-static compression conditions was investigated by finite element simulations using the commercial software ABAQUS/EXPLICIT 2022.

**Figure 4:**
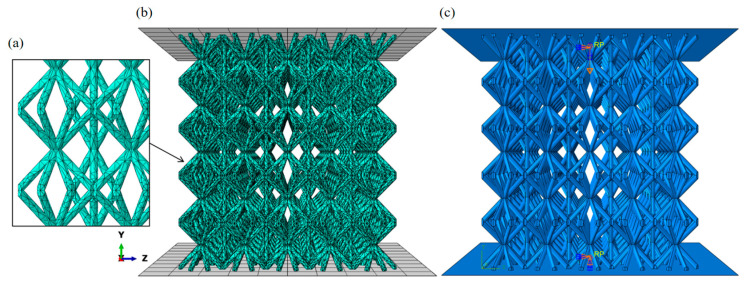
(**a**) Grid division enlarged image, (**b**) grid division schematic diagram, (**c**) *Y*-axis loading schematic diagram.

As shown in Figure 4, similar to what was mentioned earlier, for Types 1 to 5, in each of the point lattice structures of the types, the SC8 single cell adopts the same gradient design. The corresponding SC4 single cells, combined with them, adopt several gradient designs that have similar characteristics to the gradient of this SC8 single cell, and are combined to form the corresponding combined gradient point lattice structures. For example, in Type1, the SC8 single cells adopt Strategy-LE. The three combined gradient point lattice structures’ SC4 single cells adopt Strategy-LE, Strategy-ID, and Strategy-DI, similar to Strategy-LE. The numerical model consists of three parts: (1) the top loading plate, (2) the relative density gradient lattice structure modeled in five layers, and (3) the bottom support plate. The top loading and bottom support plates are set as discrete rigid bodies. Two reference points are coupled to the top plate’s lower surface and the bottom plate’s upper surface. The six degrees of freedom of the bottom plate through the bottom plate reference point are fixed. Similarly, the top plate reference point constrains five degrees of freedom except the loading direction, and a displacement of −45 mm in the loading direction U2 is set through this reference point to apply a displacement load in the Y direction downward on the top plate. A suitable meshing strategy is used to balance computational efficiency and accuracy. A general contact with a tangential friction coefficient of 0.15 was used in the numerical model. The material in the numerical model, AlSi10Mg, which is commonly used in additive manufacturing using lattice structure, was used, and its mechanical properties were characterized by the Johnson–Cook plasticity and damage model, and the values of the parameters used are given in Table 1 [42]. Additionally, the Young’s Modulus of AlSi10Mg is 75 GPa, the Poisson’s Ratio is 0.3, and the Mass Density is 2700 kg/m^3^ [40]. The model applies to most metallic materials and is widely used in similar studies [43,44,45,46,47]. The displacement and reaction force at the reference point of the roof plate are recorded to obtain the mechanical response of the numerical model in compression, and the stress–strain curves of different relative density gradient lattice structure models are plotted based on the output results.

## 4. Results and Discussion

### 4.1. Feasibility Verification of the Finite Element Simulation Results

**Figure 5:**
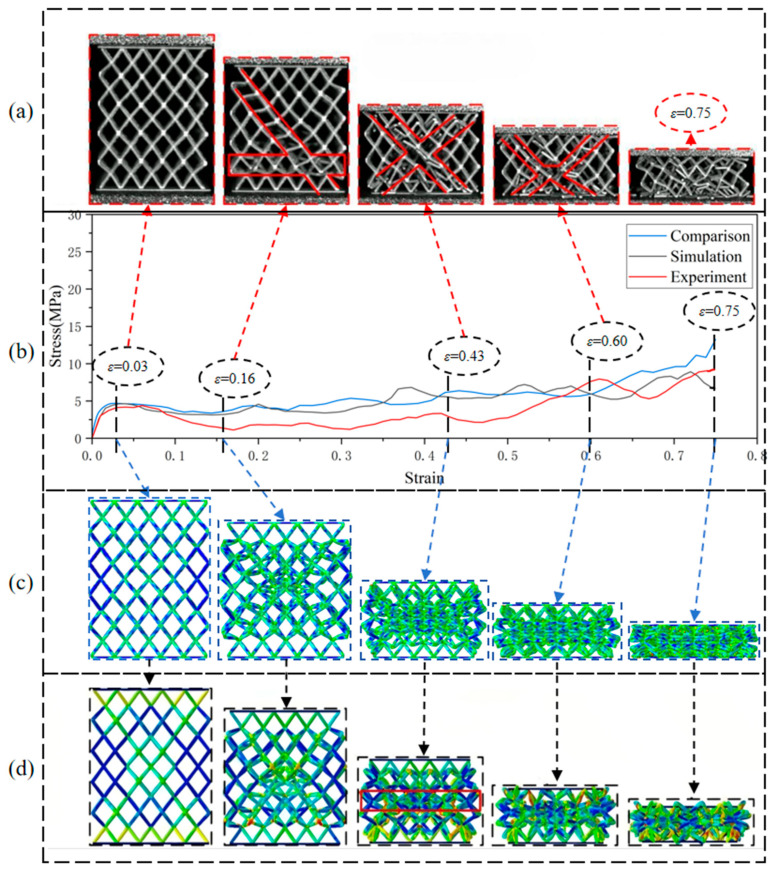
(**a**) The mechanical behavior of the N4MLU lattice structure in the literature [40]; (**b**) three stress–strain curves from the comparative simulation experiment, the numerical simulation in the literature [40], and the experiments in the literature [40]; (**c**) Mises stress contour plots of the comparative simulation experiment; (**d**) Mises stress contour plots of the numerical simulation in the literature [40].

For the N4MLU lattice structure mentioned in the literature [40], using the same material parameters, grid division strategy, and other parameter settings as those in the numerical simulation experiments of this paper, a comparative simulation experiment was conducted to study the results of the comparative simulation experiment and the results obtained in the literature. The comparison diagram is shown in Figure 5.

By comparing the stress–strain curves with the deformation behavior, it is found that the stress–strain curves of the comparative simulation experiments and the numerical simulation experiments in the literature are well fitted, and the overall trend is more consistent. The deformation behavior of the N4MLU lattice structure is highly similar to that in the numerical simulation experiments in the literature. Due to the printing defect problem of the experimental specimens mentioned in the literature, the stress–strain curves of the lattice structure obtained from the simulation have the same trend as the experimental results. Still, the difference in stress level is minor. A comparison of the results of the simulation experiment proves the feasibility of the numerical simulation experiment in this paper.

### 4.2. Compression Response and Failure Modes

The force–displacement curve of the combined lattice structure was plotted in the Visualization module of ABAQUS. After exporting the data, all the values on the *X*-axis were divided by the structure’s height (50 mm) to obtain the strain values. All the values on the *Y*-axis were divided by the cross-sectional area in the direction of the pressure applied to the structure (i.e., the area of the semi-transparent light pink square in Figure 2) to obtain the stress values. The corresponding stress–strain curve was then plotted.

**Figure 6:**
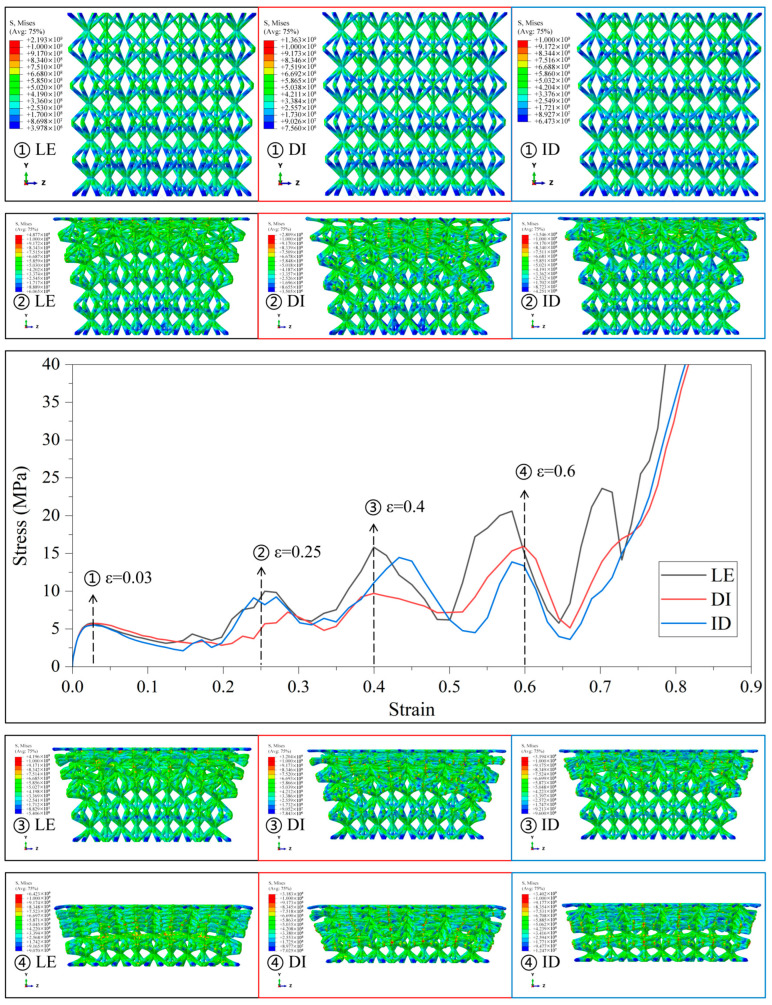
Stress–strain curves and Mises stress contour plots for the CSC8/4 lattice structures with different relative density distribution strategies in Type1.

**Figure 7:**
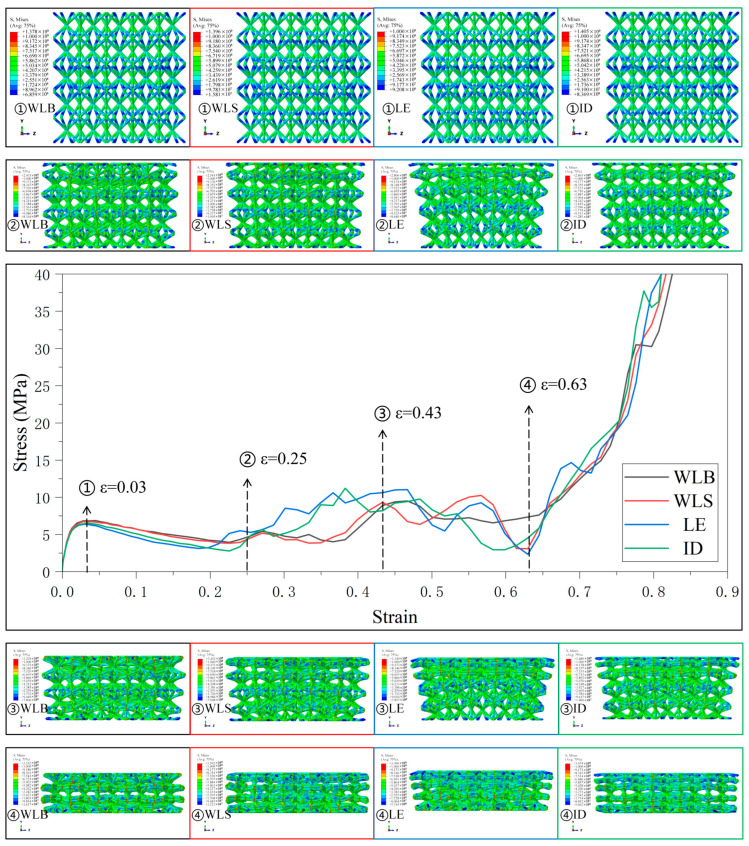
Stress–strain curves and Mises stress contour plots for the CSC8/4 lattice structures with different relative density distribution strategies in Type2.

**Figure 8:**
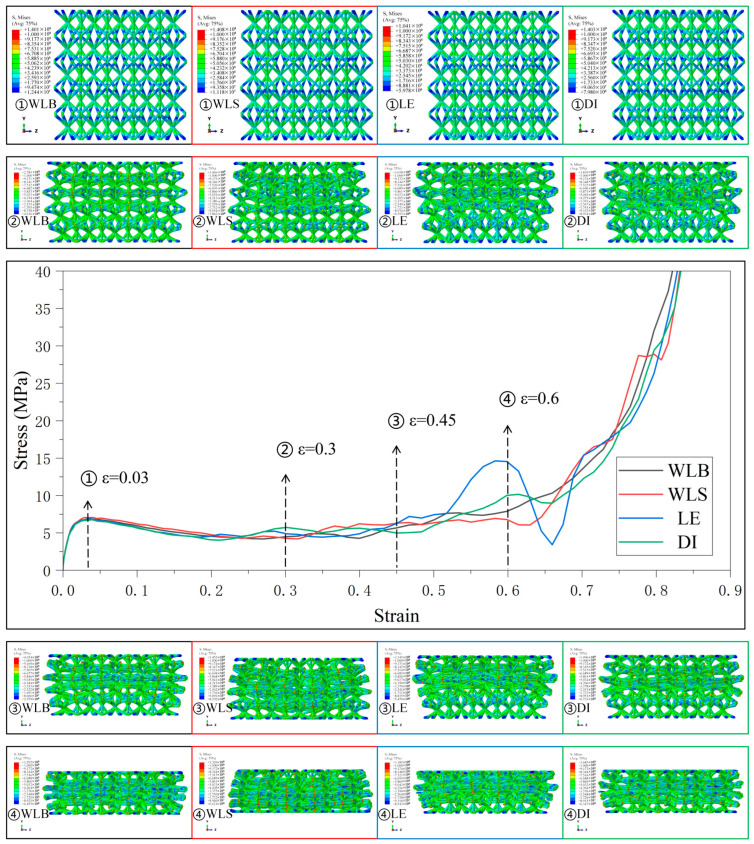
Stress–strain curves and Mises stress contour plots for the CSC8/4 lattice structures with different relative density distribution strategies in Type3.

**Figure 9:**
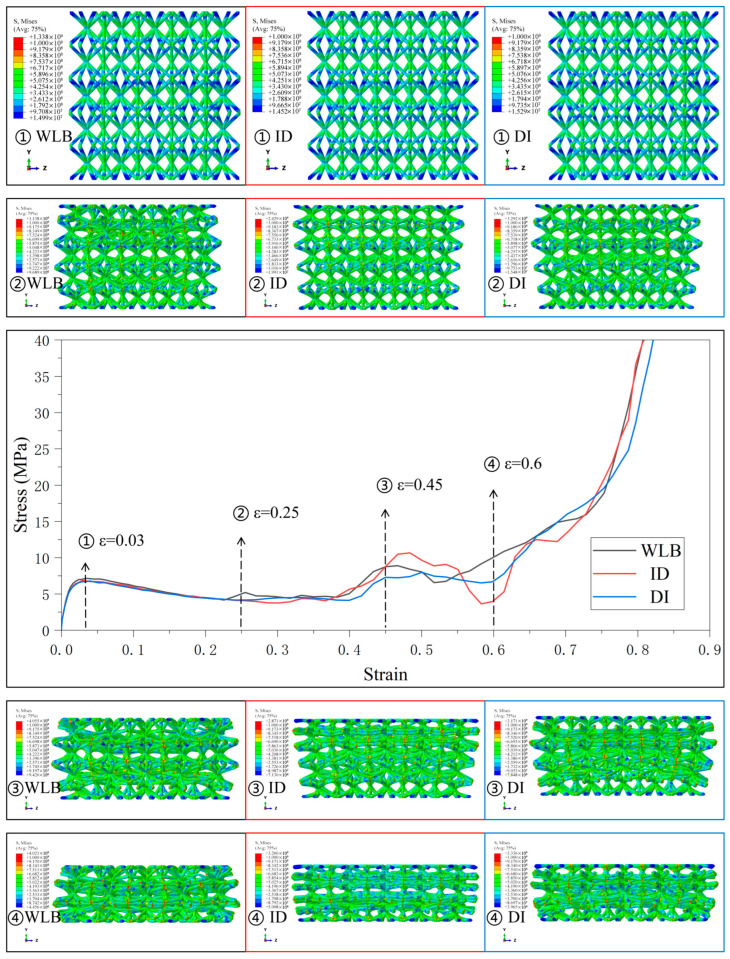
Stress–strain curves and Mises stress contour plots for the CSC8/4 lattice structures with different relative density distribution strategies in Type4.

**Figure 10:**
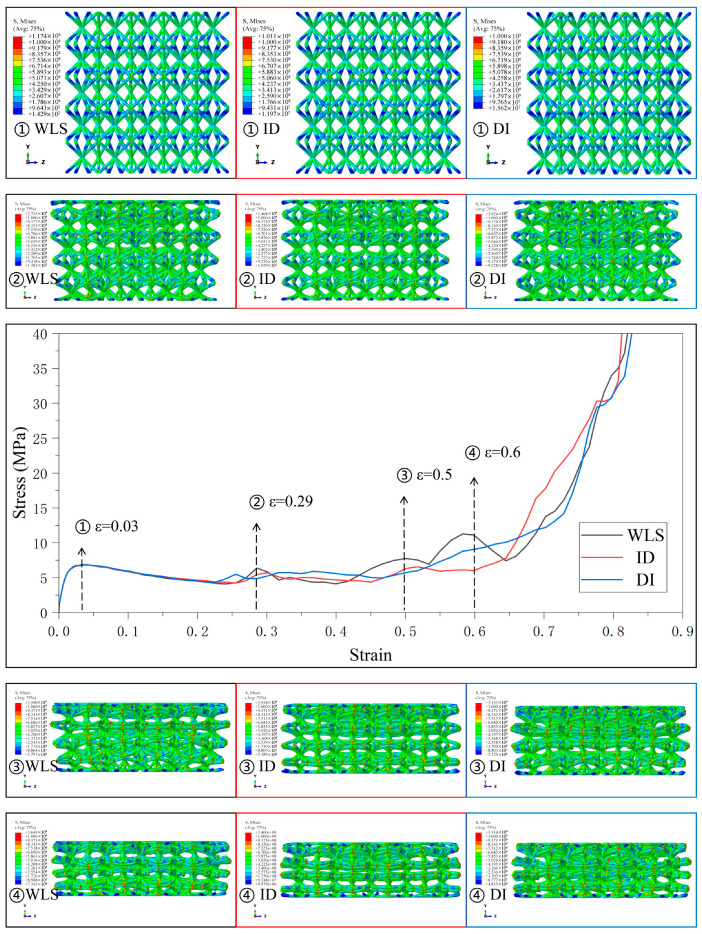
Stress–strain curves and Mises stress contour plots for the CSC8/4 lattice structures with different relative density distribution strategies in Type5.

Figure 6, Figure 7, Figure 8, Figure 9 and Figure 10 demonstrate the deformation behaviors and stress–strain curves of the lattice structure under compressive loading obtained from finite element simulation experiments with different gradient strategies. The designations of Type1 to Type5 are given according to Figure 3.

The compressive response of the lattice structure is superior to that of the two lattice structures containing only SC4 or SC8 single cells under the same conditions due to the combination of SC4 and SC8 single cells. In the following, only the mechanical behavior of the combined lattice structure CSC8/4 is analyzed in the comparison between uniform and gradient conditions.

In Type1, the SC8 cell is designed with linearly augmented gradients, and the designation given in the legend of Figure 6 represents the gradient design strategy for the SC4 cell combined with it, with similar representations are used in Figure 7, Figure 8, Figure 9 and Figure 10.

It is observed that the stress–strain curves of all lattice structures under compression can be roughly divided into four stages. The first stage is the elastic deformation stage, in which the stress increases linearly with strain in the curve. The second stage is the yield-strengthening stage, in which the stress increases nonlinearly with strain as compression proceeds and reaches the peak value, where the compressive strength of the lattice structure under small deformation can be obtained. The third stage is the transition stage, when compression continues, the stress decreases gradually from the peak value, and for different gradient designs, the curve fluctuates to varying degrees in this stage. The fourth stage is the densification stage, when the compression proceeds to a later stage, the lattice structure is almost compressed into a solid, and the stress rises sharply with strain. The stress–strain curves of the lattice structure and its deformation behavior are somewhat different under different design strategies. Therefore, various types of gradient lattice structures are discussed separately.

The mechanical responses of various gradient combinations under the Type1 strategy are given in Figure 6. It is observed that the lattice structure is deformed layer by layer from the relative density minimum under the linearly enhanced gradient design, and the larger fluctuations in the stress–strain curves arise due to the sequential collapse of the lattice structure between different layers. Since SC8 monolayers have a greater influence on the deformation behavior compared with SC4 cells, the stress–strain curves for SC8 cells with the Type1 strategy of linearly enhanced gradient design all have larger fluctuations in the transition stage. Due to the layer-by-layer deformation, the boundary lattice is tilted in the longitudinal arrangement due to the larger transverse size of the upper cells with smaller relative density and preferential deformation in the lattice structure. Obviously, in the case of SC8 cells with the same gradient design, the lattice structure composed of SC4 cells with the same linearly enhanced strategy fluctuates more drastically in the transition phase than that of SC4 cells with the “DI” and “ID” strategies, and the fluctuations in the transition phase are more intense. The gradient design of SC4 cells also affects the mechanical response of the lattice structure to some extent. However, under small deformation, the mechanical responses of the elastic deformation and yield-strengthening stages are usually determined by the deformation behavior of the weakest interlayer in the gradient lattice structure, so that under the main influence of the SC8 cells possessing linearly enhanced gradients, the first deformation and compression collapse are generated in the diameter of the top finest cell strut. There is no large gap between the elastic deformation and compressive limit of the lattice structure at the small deformation stage.

Figure 7 shows the stress–strain curves and the deformation behaviors of the four gradient combinations under the Type2 strategy. The deformation behavior of the lattice structure with different gradient combinations is still following the deformation and collapse from the weakest layer to the strongest layer, and under the four gradient combinations, since the relative density of SC8 cells in the five-layer lattice structure increases from the top layer to the bottom layer and then decreases, and is symmetrically distributed in the intermediate layer, the deformation of the lattice structure occurs at the same time in different layers, and the fluctuation in the stress–strain curves is also reduced as a result. The degree of fluctuation in the stress–strain curve is therefore reduced. Unlike the layer-by-layer deformation, when the deformation occurs almost simultaneously in different layers, the lattice structure does not have an apparent tilt in the longitudinal arrangement of the boundary lattice. However, for SC4 cells using the “ID” gradient strategy, the compression of the lattice structure starts to load on the top plate above the top layer because the linearly enhanced features in the “ID” gradient are designed in the upper part of the lattice structure. Therefore, at the beginning of loading, both SC4 cells designed with “LE” and “ID” gradients in the Type2 strategy and SC8 cells with “ID” gradients have similar relative densities to the “LE” and “ID” gradients. Both SC4 and SC8 cells using the “ID” gradient have a relative density design similar to that of the linearly enhanced gradient, and their deformation behaviors thus show a layer-by-layer deformation starting from the top layer and moving from top to bottom. In another combination, the addition of SC4 cells with the “WL” gradient, thanks to its increasing and decreasing relative density design, brings a certain degree of buffer to the interlayer deformation of the lattice structure, and thus reduces the fluctuation amplitude of the curve in the transition stage.

Figure 8 shows the four gradient combinations’ stress–strain curves and deformation behaviors under the Type3 strategy. In the Type3 strategy, the SC8 cells use the gradient strategy of “DI”, and except for the fluctuation in the middle and late stages of deformation in the combinations with the SC4 cells that use the linearly enhanced gradient, the stress–strain curves of the other combinations are smoother in the transition stage. The stress–strain curves of all combinations are relatively smooth in the transition stage, and it is obvious that the lattice structures of these gradient combinations have the tendency to deform simultaneously in different layers when subjected to pressure, and the formation of this tendency may also come from the small change in the difference in the radius of the cylindrical rods between the cells with different relative densities between the layers in the design of the gradient to a certain extent. The SC4 cell with the linearly enhanced (LE) gradient and the SC8 cell with the “DI” gradient have the characteristics of linearly enhanced relative densities in the lower part of the lattice structure, and the compressive loading starts from the top, so the stress–strain curve fluctuates due to the tendency of layer-by-layer deformation in the middle and later part of the deformation of this combination. Therefore, the stress–strain curve fluctuates in the latter part of the deformation due to the tendency of layer-by-layer deformation. For the combination lattice structure with the “DI” gradient, since the loading starts from the top, and the linearly enhanced relative density feature is in the lower part of the structure, the deformation shows the trend of simultaneous deformation between different layers, instead of the trend of layer-by-layer sequential change, and the stress–strain curve does not fluctuate greatly.

Figure 9 and Figure 10 present the stress–strain curves and mechanical behaviors of the lattice structures using SC8 cells with the wave gradient and several combinations of SC4 cells with different gradients under Type4 and Type5 strategies. The deformation characteristics are similar to those previously described, and the deformation behaviors of the lattice structures with varying combinations of gradient follow the deformation trend and collapse from the weakest to the strongest layer, accompanied by a small portion of simultaneous deformation of the interlayers to some extent. The stress–strain curves, therefore, contain several fluctuating phases and several smooth phases.

**Figure 11:**
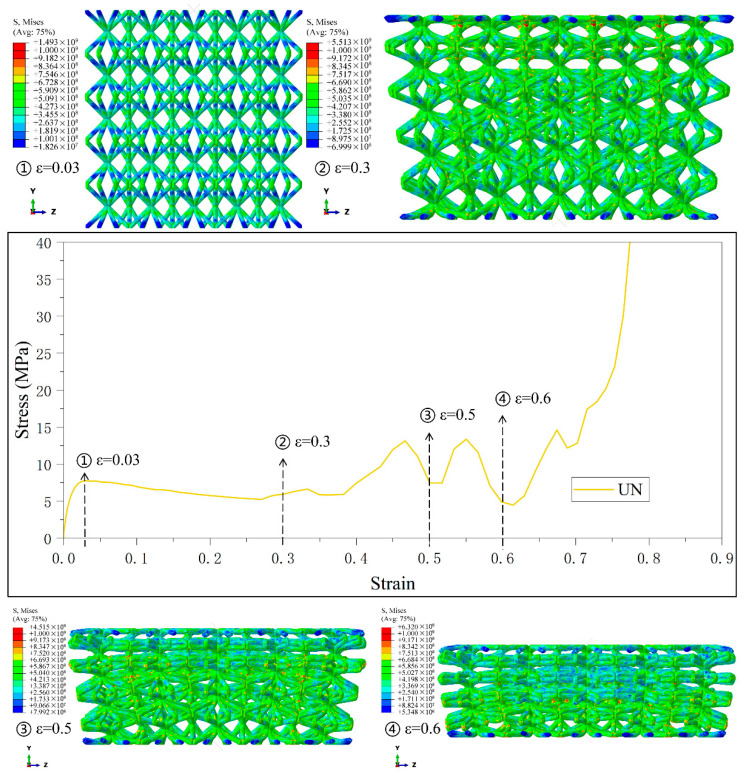
Stress–strain curves and Mises stress contour plots for the CSC8/4 lattice structures with different relative density distribution strategies in Type6.

The mechanical response of the uniformly combined lattice structure under the Type6 strategy is shown in Figure 11. Since the cylindrical rods in the single cell of this lattice structure have identical diameters, the structure exhibits the mechanical behavior of simultaneous compressive deformation of the single cell in different layers when the strain is slight, and the stress–strain curves are relatively flat in this stage. The deformation mode of layer-by-layer deformation is also shown when the strain is significant, and the stress–strain curves in this stage also have the typical characteristics of fluctuation due to the layer-by-layer deformation. The mechanical response of the uniform combination lattice structure of the Type6 strategy reflects the mechanical properties of the CSC8/4 combination lattice structure that is independent of the gradient.

### 4.3. Mechanical and Energy Absorption Properties

The elastic deformation and yield-strengthening stages determine the lattice structure’s specific modulus and specific compressive strength when the structure is deformed under pressure. Their magnitudes are directly related to the mechanical properties of the weakest layer in the multilayer gradient lattice structure, i.e., the layer with the smallest relative density. The calculation formulas are, respectively, as follows:

(4) $$\mathrm{Specific}\;\mathrm{Modulus}=\frac{E}{\rho }=\frac{E}{\rho_{\mathrm{material}}\;\bar{\rho }}$$

(5) $$\mathrm{Specific}\;\mathrm{Compressive}\;\mathrm{Strengths}=\frac{\sigma_{b}}{\rho }=\frac{\sigma_{b}}{\rho_{\mathrm{material}}\;\bar{\rho }}$$

where E is the elastic modulus of the lattice structure, σ_b_ is the compressive strength of the lattice structure, and ρ is the density of the lattice structure, which is the product of the density of the material and the relative density of the lattice structure.

**Figure 12:**
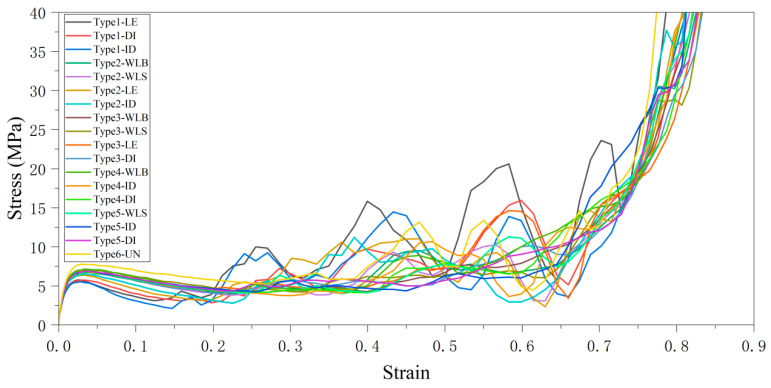
Stress–strain curves of combined gradient lattice structure vs. uniform structure.

The specific modulus and compressive strength of the lattice structure with different relative density gradient strategies are summarized in Table 2. Obviously, the radius of the cylindrical rods in the single unit cell of the weakest layer in the gradient lattice structure is smaller than that of the cylindrical rods in the uniform lattice structure. The specific modulus and specific compressive strength of the combined gradient lattice structure have different degrees of attenuation compared to that of the uniform lattice structure, and the stress–strain curves of all the proposed combined gradient lattice structures compared to the uniform structure are shown in Figure 12. This phenomenon is also reflected in other studies [40,48,49]. Among them, the SC8 single cells adopt the Type1 scheme of the linearly enhanced relative density gradient, which has the weakest performance among the proposed combined gradient lattice structures. Due to the linearly enhanced gradient design feature contained in its lattice structure, it exhibits poor mechanical properties when subjected to compressive loading, starting from the layer with the lowest relative density, and collapsing under compression sequentially layer by layer with the growth of the relative density of the layers. At the same time, the combined gradient lattice structures containing the wave gradient design feature all show better mechanical properties than the uniform structures. Thanks to the relative density design of the increasing and decreasing wave gradient, the interlayer deformation of the lattice structure under compressive load is buffered to a certain extent, resulting in more excellent mechanical properties.

The multilayer BCC lattice structure possesses a long energy absorption platform and promising applications in energy absorption. Therefore, the energy absorption capability of the proposed multilayer lattice structure is evaluated by two metrics, Specific Energy Absorption (SEA) and crash load efficiency (CLE), which are representative in the field of energy absorption property evaluation. Specific Energy Absorption (SEA) is commonly used to describe the energy absorbed per unit mass of a material during deformation. Crash load efficiency (CLE) is an index used to evaluate a structure’s or material’s energy absorption efficiency during a collision. Its calculation formula is usually related to the energy absorption and load transfer characteristics. The calculation formulas of SEA and CLE given in this paper are as follows:

(6) $$\mathrm{SEA}=\frac{\int_{0}^{\varepsilon_{d}}\sigma (\varepsilon )d\varepsilon }{\rho }$$

(7) $$\mathrm{CLE}=\frac{\sigma_{p}}{\sigma_{e}}=\frac{\frac{1}{\varepsilon_{2}-\varepsilon_{1}}\int_{\varepsilon_{1}}^{\varepsilon_{2}}\sigma (\varepsilon )d\varepsilon }{\sigma_{e}}$$

where ρ is the density of the lattice structure, ε_d_ is the strain at the onset of lattice structure densification, σ_p_ is plateau stress, and σ_e_ is initial peak stress, and platform strains are in the range of ε_1_ to ε_2._ The SEA and CLE values for various combinations of gradient lattice structures are shown in Table 3.

**Figure 13:**
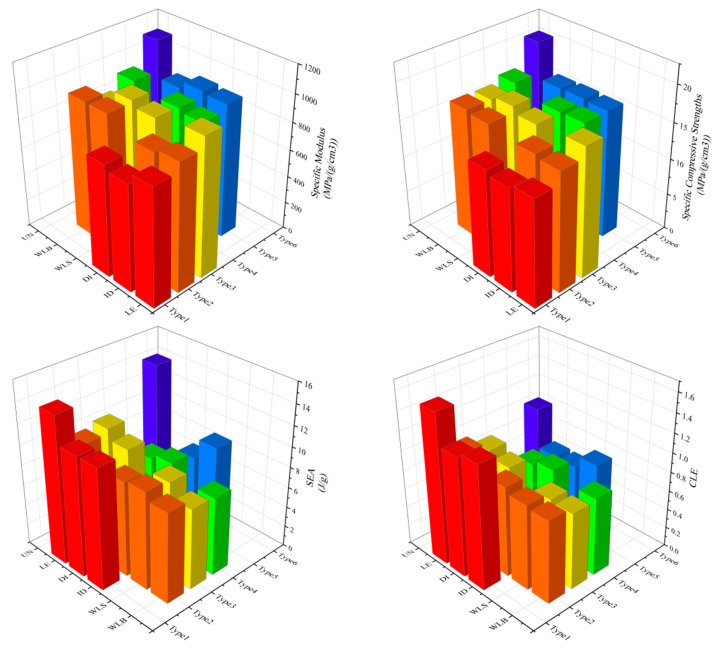
Column comparison of specific modulus, specific compressive strength, SEA, and CLE values for various combinations of gradient lattice structures.

Unlike the corresponding mechanical properties, the linearly enhanced gradient strategy, due to its more stable and straightforward layer-by-layer deformation behavior when subjected to compressive loading, has the most outstanding energy absorption capability, even surpassing the energy absorption properties of the uniform lattice structure when the “LE” gradient strategy is used for both the combined gradients, with a 6.36% increase in SEA value and a 61.6% increase in CLE value compared to that of the uniform structure. The wave-like gradient strategy, which has the most superior mechanical properties, has the worst energy absorption properties due to its delayed deformation under pressure. Column comparison plots of specific modulus, specific compressive strength, SEA, and CLE values for various combinations of gradient lattice structures are shown in Figure 13.

In addition, the relative density distribution of the multilayer lattice structure can be carefully adjusted to achieve ideal mechanical response and energy absorption characteristics, which is essential for practical engineering.

### 4.4. Numerical Simulation Results of Combined Gradient Lattice Structure and Nanomechanical Mechanism

In our BCC lattice model, collapse is primarily driven by the buckling of inclined struts and plastic yielding at nodal regions (where multiple struts intersect). From a nanomechanical perspective, the critical buckling stress of struts (determined by slenderness ratio and elastic modulus) mirrors the instability behavior of nanoscale beams [50]. Stress concentration at nodes may further initiate localized yielding, propagating collapse layer by layer. This sequential failure mode, coupled with the relative density gradient design (e.g., low-density regions collapsing first), collectively governs the energy absorption performance.

Although crack propagation is not explicitly modeled, high stress concentration at nodes (the areas presented in red in Figure 6, Figure 7, Figure 8, Figure 9, Figure 10 and Figure 11) may reach the theoretical strength limit of the material, serving as potential sites for crack initiation. Insufficient energy dissipation during collapse (e.g., limited post-buckling plasticity) could convert residual energy into crack-driving force. This mechanism is closely tied to the relative density gradient in BCC lattices: high-density regions mitigate stress spikes by delaying collapse, thereby reducing crack susceptibility [51].

The macroscopic energy absorption is characterized by the Johnson–Cook (JC) constitutive model, which couples strain hardening, strain rate effects, and thermal softening, effectively capturing nanoscale dissipation mechanisms (e.g., dislocation multiplication and thermally activated slip) under dynamic loading. Simulations demonstrate that prioritized collapse in low-density regions extends the stress plateau, enhancing energy absorption efficiency through progressive layer collapse. The damage parameters in the JC model (e.g., fracture strains D1-D3) further reveal the crack initiation threshold at nodal regions, collaboratively governed by the relative density gradient and material failure criteria.

## 5. Conclusions

A BCC combined single-cell lattice structure, CSC8/4, was designed, and several relative density gradient models were developed based on its structure. Finite element simulations were performed using the commercial software ABAQUS/EXPLICIT, and the simulations were subjected to quasi-static compression tests. The mechanical responses, deformation modes, and energy absorption properties of the lattice structures with different combinations of gradients were systematically investigated. And the following conclusions are drawn:

(1) The relative density gradient strategy introduced in the combined single-cell lattice structure has stronger designability than the uniform lattice structure, and a total of 18 different combinations of relative density gradient designs are proposed, which significantly enhances the adaptability of the BCC lattice structure in practical complex application scenarios. It found that when applied to composite lattice structures, the proposed wave-like gradient design leads to a notable improvement in mechanical properties. Among the various gradient strategies examined, several have achieved mechanical performance close to that of uniform lattice structures. To some extent, this approach mitigates the common drawback of gradient lattice structures—where the relative density of the weakest layer is consistently lower than the interlayer relative density of uniform lattice structures—resulting in varying degrees of mechanical performance degradation compared to their uniform counterparts.

(2) The relative density gradient design scheme can sacrifice its stiffness and strength to enhance the energy absorption properties of the lattice structure. The proposed linearly enhanced gradient strategy (Strategy-LE) has lower values of specific modulus and specific compressive strength when the lattice structure is subjected to compressive loading, but has the highest SEA and CLE values simultaneously. The proposed wave gradient strategy, on the other hand, exhibits excellent mechanical properties but has poor energy absorption characteristics.

In summary, the proposed combined gradient lattice structure can exhibit superior energy absorption compared to the conventional uniform lattice structure. Compared with the existing relative density gradient lattice structure, it can exhibit more excellent mechanical properties. It has significant potential for practical applications in aerospace, biomedicine, architectural engineering, and rail transportation. Future research should focus on the optimal design of the structure, including the distribution of the relative density gradient and the configuration of single cells in the lattice structure to meet the requirements of specific applications.

## Figures and Tables

**Table 1:** Parameters of the Johnson–Cook model.

Johnson–Cook plasticity	A /MPa	B /MPa	n	m	Melting Temp/K	Transition Temp/K
	331.17	579.647	0.99	0.945	843	298
Johnson–Cook damage	d1	d2	d3	d4	d5	Reference Strain Rate
	0.04704	1.155	−0.841	−0.042	0	1

**Table 2:** Specific modulus and specific compressive strength of various combinations of gradient lattice structures.

		Specific Modulus (MPa/(g/cm^3^))	Specific Compressive Strengths (MPa/(g/cm^3^))
Type1	LE	860.656	14.685
	DI	816.549	14.634
	ID	778.641	14.115
Type2	WLB	990.742	17.552
	WLS	989.371	17.244
	LE	928.275	16.292
	ID	900.520	16.659
Type3	WLB	885.015	17.461
	WLS	1002.118	18.050
	LE	1010.233	17.698
	DI	962.902	17.246
Type4	WLB	989.048	18.293
	ID	930.715	17.454
	DI	952.321	17.344
Type5	WLS	942.463	17.570
	ID	980.051	17.511
	DI	993.995	17.660
Type6	UN	1075.480	19.890

**Table 3:** SEA and CLE values for various combinations of gradient lattice structures.

		SEA (J/g)	CLE
Type1	LE	14.411	1.559
	DI	11.748	1.238
	ID	11.771	1.287
Type2	WLB	8.820	0.861
	WLS	9.556	0.884
	LE	10.729	1.059
	ID	9.306	0.941
Type3	WLB	7.874	0.793
	WLS	9.238	0.810
	LE	11.157	0.964
	DI	10.168	0.897
Type4	WLB	7.675	0.810
	ID	8.802	0.867
	DI	8.212	0.810
Type5	WLS	10.190	0.903
	ID	8.163	0.775
	DI	6.464	0.781
Type6	UN	13.549	0.965

## Data Availability

The original contributions presented in this study are included in the article. Further inquiries can be directed to the corresponding author.

## Nomenclature

A	Yield stress (MPa)
B	Strain hardening coefficient (MPa)
C	Strain rate hardening coefficient
n	Strain hardening exponent
m	Thermal softening exponent
d_1_	Initial failure strain
d_2_	Exponential factor
d_3_	Triaxiality factor
d_4_	Strain rate factor
d_5_	Temperature factor
σ_e_	Initial peak stress
σ_p_	Plateau stress
σ_b_	Compressive strength of the lattice structure
ε_d_	Strain at the onset of lattice structure densification
ρ	Density of the lattice structure
δ_SC_	Relative error
E	Elastic modulus of the lattice structure
l	Length of an inclined strut
V_cylindricity_	Total volume of cylindrical rods within the single cell
V_space volume_	Volume occupied by the single cell in space
θ	Angle between the inclined strut and the horizontal plane in a single cell
